# Supplementary material for: UVB-Pretreatment-Enhanced Cadmium Absorption and Enrichment in Poplar Plants
Source: Int J Mol Sci. 2022 Dec 20;24(1):52. doi: 10.3390/ijms24010052 (PMC9820001; doi:10.3390/ijms24010052)
Supplement: Supplementary file 1 [file ijms-24-00052-s001.zip › ijms-2069749-supplementary.pdf]

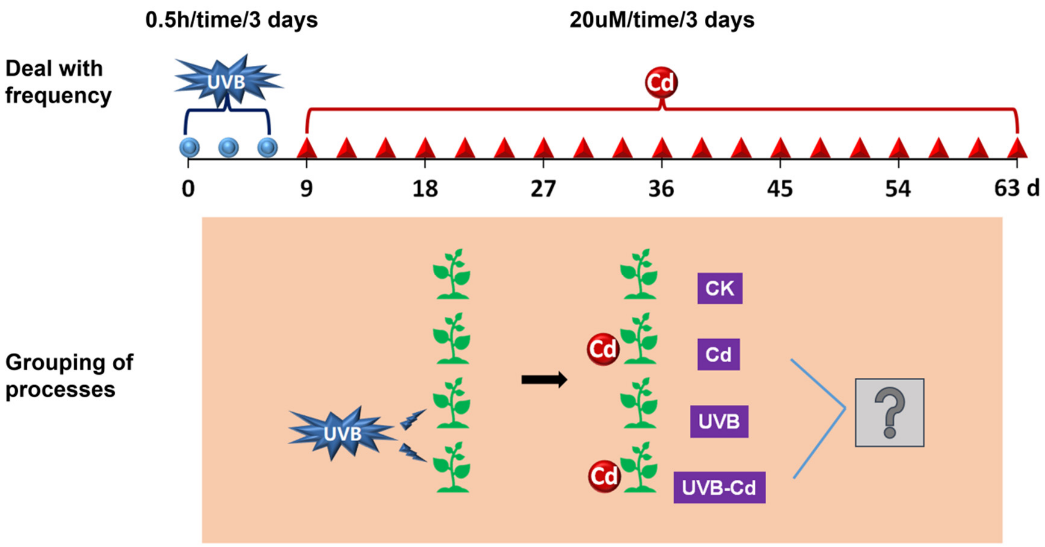

Figure S1. Schematic diagram of test design

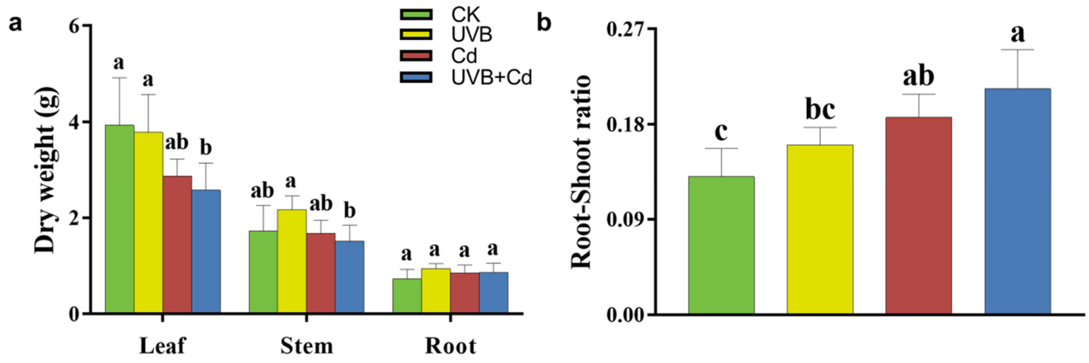

Figure S2. Dry weight of different tissues (a) and the root-shoot ratio (b) of plants under different treatments. Values are means ± SE. Different letters indicate significant differences among treatments at $p < 0.05$ according to a post hoc Duncan test followed by a one-way ANOVA.

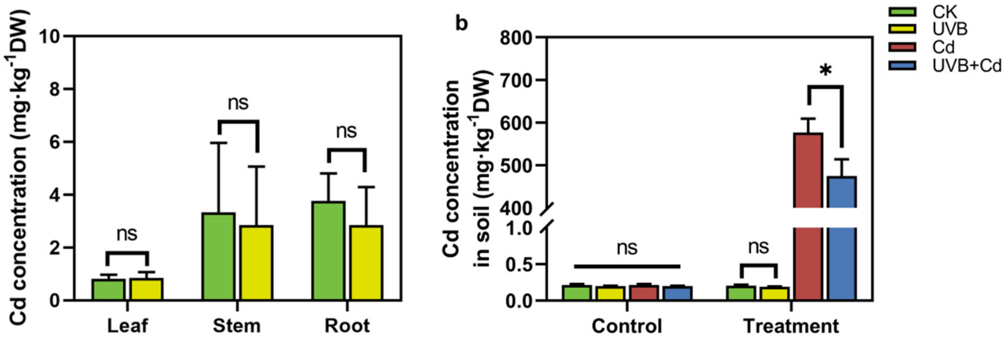

Figure S3. Cadmium content under different treatments in different plant tissues (a) and soil (b). Values are means ± SE. Asterisks denote significant differences: * represent $p < 0.05$.

Table S1. RT-qPCR primer

Name	Gene ID	F-primer	R-primer
PtrActin	Potri.001G309500	CCCATTGAGCACGGTATTGT	TACGACCACTGGCATACAGG
PtrUBQ	Potri.007G123300	AACAGCTTGAGGATGGACGA	TTTGCTGGTCCGGAGGGATA
ABCC1	Potri.011G091200	ATTGTTCAGGGCTTGTTTGGA	TATGTTGGCGTGCCTCTCAG
ABCC2	Potri.004G034800	GTTCGGTGCGCTTTGTGAAT	ATTGCAGCCACCAGAGTTGA
NRAMP1	Potri.001G044900	CCACAGTTCATTGCCAGCAC	GTTTTCCAGTGACAACGCCC
F3H	Potri.005G113900	TTTTGCTTTGCCACCGGAAG	AGTCTCTGGTGCGAATTGGG
MTP1	Potri.014G106200	CCACACCCCGCCAATCTTAT	AGGCCAAATGCAGCAACAAG
ZIP2	Potri.009G034600	CTATGGGGCTTGCATGTGGA	ACTGCAATAACTCCCACCCC
ZIP4	Potri.018G053300	GAGAAGGCAGCAGTAGAGGC	GTGAAAGCTTAGGGCAGCCA
ZIP6	Potri.009G074100	TGTTTCGCGGCAGGAGTAAT	CCCACCATTTCCTCCTGTGT
PCS1	Potri.014G195800	CCGGGTGAATTGAGGGGAAA	CACACCGTACCTGAGCCAAT
MYB12	Potri.010G141000	ACAAGGAGGACAACGAAGGC	CAACCGAGGATGCACAAACC
GSH	Potri.005G038100	TTCATCCCCTCTTTCACCGTC	TGATACTGTTCCTGGGTTTGC
YSL1	Potri.001G084400	AGAGAGAATGGTGTGGTGGC	GGGGGAGGTGGATGTCATTT
CAX1	Potri.001G251200	CACAGGCTGATCGGGTCTC	TTTGAAACCGAGCACTTGACG

CAX2	Potri.001G469800	TGCACTACACACACACGGAG	CGGAGTCTCGTCGTCATCATT
CAX3	Potri.006G099900	TCACCACAGCCTTCACCTTAC	AGCTGTGATGACTCCGACCT
CAX4	Potri.009G045800	ATGTGTTGTTGTTGCGTGGA	CGCTAGCTAGGAACTTACTC
CAX5	Potri.010G249200	GCGTTGTTGTTGGATGGTGT	ATGCCACCACTAGCACAGAG
CAX6	Potri.011G166900	GGGGCTACTCTTTCCTGCTG	GGAGTCTCATCATTGTCGCCT
CAX7	Potri.016G115500	GGATTTCTGCAGCCTCCGTT	ATCGCCATCTTCCACGGATTC
